# Development of oculomotor digital biomarkers using clinical examinations as “Ground Truth”

**DOI:** 10.3389/fnhum.2025.1556451

**Published:** 2025-05-21

**Authors:** Brittany Trotter, Melissa Hunfalvay, Nicholas P. Murray, Greg C. Mathews, Frederick Robert Carrick

**Affiliations:** ^1^East Carolina University, Department of Kinesiology, Greenville, NC, United States; ^2^RightEye LLC, Bethesda, MD, United States; ^3^Biological Engineering, Massachusetts Institute of Technology, Cambridge, MA, United States; ^4^The Brain Health Center of Maryland, Silver Spring, MD, United States; ^5^College of Medicine, University of Central Florida, Orlando, FL, United States; ^6^Department of Neurology, The Carrick Institute, Canaveral, United Kingdom; ^7^Centre for Mental Health Research in Association with the University of Cambridge, Cambridge, United Kingdom; ^8^Burnett School of Biomedical Science, University of Central Florida, Orlando, FL, United States

**Keywords:** eye tracking, oculomotor behavior, saccade, smooth pursuit, clinical examination, digital biomarkers

## Abstract

**Objective:**

The purpose of this study was to assess the validity of six computerized eye-tracking tests against a clinician-administered oculomotor exam.

**Methods:**

A total of 53 participants completed the horizontal random saccade (HRS), circular smooth pursuit (CSP), horizontal smooth pursuit (HSP), vertical smooth pursuit (VSP), horizontal saccade (HS), and vertical saccade (VS) oculomotor tests on the computerized system. A board-certified neurologist with 16 years of experience also conducted an oculomotor examination to mirror eye movement patterns.

**Results:**

Data analysis included a series of single-block logistic regressions to examine the scoring of the six eye-tracking tests (RightEye, LLC) to predict clinician-rated eye movement classifications (i.e., normal or abnormal). The computerized battery demonstrated concurrent validity for each of the six oculomotor tests as they significantly predicted the neurologist’s classification. The sensitivity and specificity of the six eye-tracking tests ranged from 70.4 to 93.5% and from 84.6 to 90.5%, respectively. The diagnostic accuracy of the computerized tests ranged from good (78.8%) to excellent (92.3%). The area under the curve (AUC) analysis for the eye-tracking tests yielded values ranging from 0.734 (VSs) to 0.921 (HRSs).

**Conclusion:**

The results suggest that each of the six computerized eye-tracking tests accurately distinguished between normal and abnormal oculomotor movements.

## Introduction

The visual system is responsible for detecting colors, shapes, and motion and contributes to attention and spatial awareness through millions of tracts in the brain (Gilbert, 2013; Jones, 1975; Lynch et al., 1977). The oculomotor system controls eye positioning and fixation and coordinates eye movement toward a directed target through six functions: saccades, smooth pursuits, fixation, vergence, vestibular-ocular reflex (VOR), and optokinetic reflex (OKR) (Sharpe and Wong, 2005). With 20%–30% of the cerebral cortex involved in visual processing and relaying visual information, it is understandable that systemic stressors (e.g., diabetes), trauma (e.g., concussion), vitamin deficiency (e.g., retinoic acid), or neurodegeneration (e.g., Alzheimer’s disease) may present clinically during neurological, ophthalmologic, or optometric examination—even in the absence of other hallmark signs or symptoms of the disease (Bardsley et al., 1993; Berson et al., 1993; Boucart et al., 2014; Ciuffreda et al., 2014; Van Essen, 2004).

Meaningful testing of visual function requires exceptionally high granularity, as many oculomotor systems are closely interrelated. For example, in concussion patients with a receded near point of convergence distance, the receded distance was not entirely diagnostic of isolated convergence insufficiency (Raghuram et al., 2019). Instead, it reflected isolated accommodative disorders (39%), isolated convergence insufficiency (8%), a combination of convergence insufficiency and concurrent accommodative disorders (34%), normal binocular vision (6%), or other oculomotor disorders (13%). Furthermore, some abnormal eye movements may be brief or subtle and difficult for less-trained practitioners to identify and subsequently treat. Therefore, it is critical to have technologies that can reliably and accurately quantify eye movements, track performance over time, and compare patients to other normative data to provide optimal patient care.

Eye tracking is an emerging technique that is receiving considerable attention as it can generate many of the above-mentioned metrics in a relatively brief examination. RightEye, LLC (Bethesda, MD, United States) utilizes eye-tracking technology that has demonstrated good to excellent test–retest reliability [intraclass correlation coefficients (ICCs) = 0.70–0.95] and acceptable to excellent internal consistency (Cronbach’s *α* = 0.70–0.95) for dynamic visual acuity testing (Murray and Hunfalvay, 2017). A battery of its oculomotor tests was also examined and included horizontal random saccades (HRSs), circular smooth pursuits (CSPs), horizontal smooth pursuits (HSPs), vertical smooth pursuits (VSPs), horizontal saccades (HSs), and vertical saccades (VSs) (Murray et al., 2019). Test–retest reliability of individual metrics within the oculomotor tests ranged from 0.4 (i.e., unacceptable) to 0.9 (i.e., excellent), and a cluster analysis yielded distinct age groups that were used to develop norms. However, these metrics have not been validated against an oculomotor exam. Therefore, the purpose of this study was to assess the validity of six computerized eye-tracking tests (RightEye, LLC) against an oculomotor examination performed by a board-certified neurologist.

## Materials and methods

### Setting and study population

Participants were recruited for this study through advertisements placed on the Internet, social media, bulletin boards, and word of mouth. Participants were included if they responded to the recruitment materials, met the inclusion criteria, did not exhibit any exclusion criteria, and provided consent to participate in the study. Due to potential known confounds within the diagnosis and eye-tracking measures, participants were excluded from the study for participation if they met any of the following pre-screening conditions: known neurological disorders (e.g., Parkinson’s disease and cerebral palsy); concussion within the last 10 years; vision-related issues that prevented successful eye-tracking calibration of all 9-points, such as extreme tropias (Niehorster et al., 2018; Renard et al., 2015; Han et al., 2010), phorias (Han et al., 2010; Kooiker et al., 2016), static visual acuity of worse than 20/400 (Niehorster et al., 2018), nystagmus (Niehorster et al., 2018; Kooiker et al., 2016) cataracts, and eyelash impediments (Holmqvist and Nyström, 2011); or reported recreational drug use or alcohol consumption within 24 h of testing. All participants reported no prior experience with any eye-tracking technologies. This research was reviewed by the University Institutional Review Board (IRB) and conformed to the principles and applicable guidelines for the protection of human subjects in biomedical research.

### Observation procedures

The nature of the study was explained to participants, and all participants provided written consent to participate. After completing informed consent, participants were asked to complete the pre-screening questionnaire. If any of the pre-screening questions were answered positively, the participant was excluded from the study. Participants who passed the pre-screening were then randomly assigned to the order of testing using a 1:1 ratio, either with the oculomotor examination first or the computerized eye-tracking tests first. Once the participants completed the first set of tests (oculomotor examination or computerized eye tracking), they moved to the next test area and completed the remaining tests. After the completion of the study, the participants were provided with a USD$ 25 gift card for their participation in the study and were debriefed before leaving. The researchers and clinicians were blinded to the participants’ computerized eye-tracking performance during testing, which was only revealed during data analysis.

### Measures

**Figure 1:**
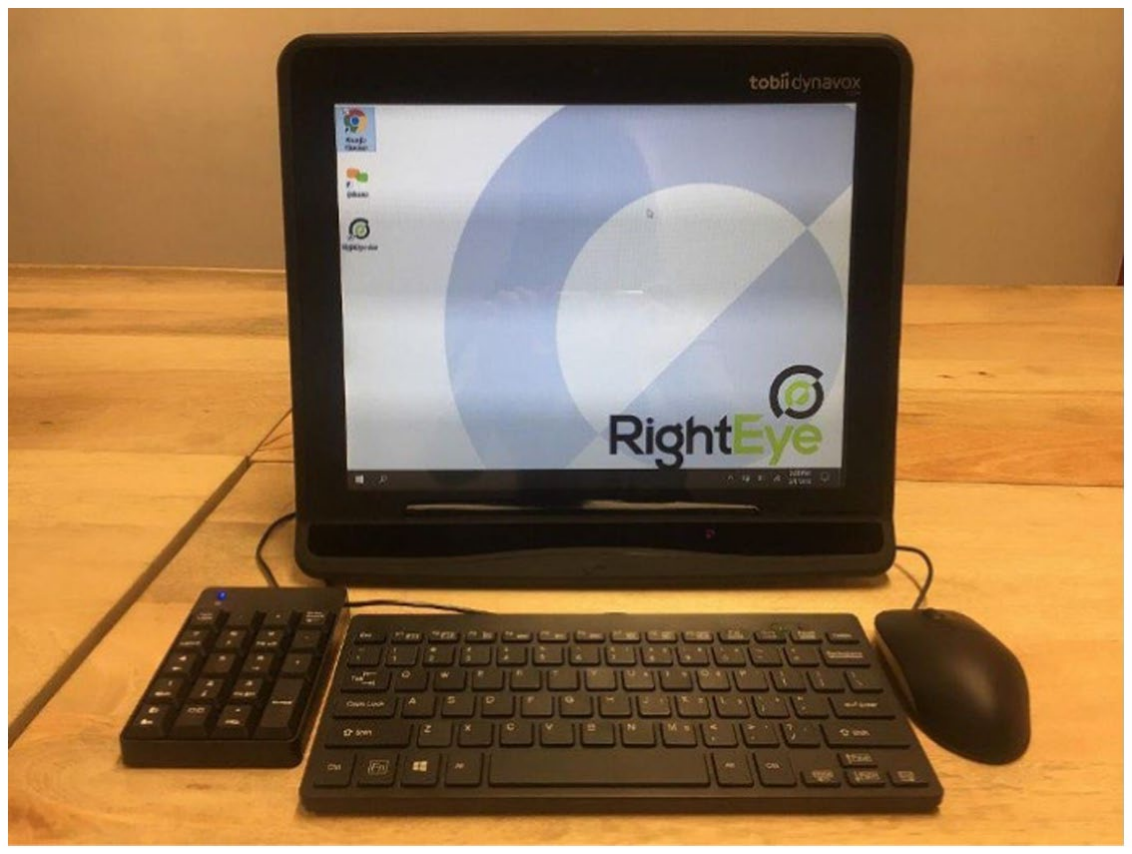
RightEye vision testing system: Tobii Dynovox i15 all-in-one device used for the computerized apparatus.

The participants were seated in a height-adjustable, stationary chair placed next to a desk inside the laboratory (Figure 1). All tests were completed by a research staff member who had received and passed the RightEye training, education, and protocol procedures before data collection. The participants were asked to look at a Tobii Dynavox, i15 all-in-one system. The screen dimensions were 12″ wide × 9″ high and placed at a distance between 55 and 60 cm from the participant’s face. The system was fitted with a Tobii 90 Hz remote eye tracker connected to the computer with a wired keyboard and mouse.

Qualified participants who successfully passed the 9-point calibration procedure completed the computerized eye-tracking test battery. Before each test, written instructions and computer animations were provided to demonstrate appropriate eye movement. Participants who needed vision correction at 55–60 cm from the screen were permitted to use correction. For each test, the participants were asked to follow the stimuli presented on the screen as “accurately as possible with your eyes.” Participants completed the horizontal random saccade (HRS), circular smooth pursuit (CSP), horizontal smooth pursuit (HSP), vertical smooth pursuit (VSP), horizontal saccade (HS), and vertical saccade (VS) tests for the computerized eye-tracking test battery (Murray et al., 2019). Additional information on sub-variables was previously described for each computerized test. Specific to the HS and VS tests, participants completed three guided practice repetitions to ensure comprehension before testing began.

### Clinician-administered oculomotor examination

Under the supervision of a board-certified neurologist with 16 years of clinical experience, participants were instructed to follow the neurologist’s instructions. The instructions included the following steps: “follow the tip of my finger” in a slow, circular clockwise fashion (CSPs), then in a horizontal direction (i.e., left-and-right) (HSPs), and later in a vertical direction (i.e., up-and-down) (VSPs). The neurologist then conducted horizontal and vertical saccade tests, asking the participants to “move your eyes as quickly and accurately as possible between my index fingers when you hear me click.” The index fingers were approximately 20 cm apart, centered on the participant’s midline, eliciting a visual angle of 10 degrees in each direction, which mirrors the computerized eye-tracking saccade tests. The midpoint was approximately aligned with the participants’ tip of the nose for movements in the horizontal plane (HS) and then in the vertical plane (VS). The neurologist then asked participants to follow his finger as it “jumped” randomly across a horizontal plane (HRSs). The participants completed one set of five trials for each test. These eye movements were intended to reflect similar oculomotor patterns evaluated by the computerized eye-tracking system. Importantly, the neurologist did not produce a clinical diagnosis.

A trial “failure” was defined as a loss of fixation along the circular, horizontal, or vertical course of the smooth pursuit tests. For the horizontal and vertical saccade tests, “failure” was defined as either an overshoot or undershoot of the target. If a participant “failed” at least three trials (out of five) on any one pursuit or saccade test, the test was classified as “abnormal.” An overall classification of abnormal oculomotor function was defined as receiving an “abnormal” result for at least three of the five tests (i.e., circular smooth pursuit, horizontal smooth pursuit, vertical smooth pursuit, horizontal saccade, and vertical saccade). The overall oculomotor function classification was compared to the HRS computerized test performance.

### Statistical analysis

A series of single-block logistic regression analyses examined the ability of the computer-generated scoring of the six eye-tracking tests (i.e., HRSs, CSPs, HSPs, VSPs, VSs, and HSs) to predict clinician-determined eye movement classifications (i.e., normal or abnormal) for each of the corresponding tests or overall oculomotor function. Participants were randomly assigned to each test to reduce order effects; however, to ensure there was no effect of the order, a series of ANOVAs were performed to examine computerized eye-tracking performance when tested first or second for all computer sub-variables. For each logistic regression, the analysis included χ^2^ statistics, Nagelkerke *R*^2^ values, Hosmer–Lemeshow tests, and Wald statistics. The χ^2^ tests compared the log-likelihoods of the baseline model (no eye-tracking variables included) and the new model (all eye-tracking variables included for each type, i.e., HRSs, VRSs, HSs, VSs, and CSPs). Nagelkerke *R*^2^ values quantified the variance in clinical diagnosis explained by the eye-tracking tests. Hosmer–Lemeshow tests examined the goodness-of-fit of the new models. Wald statistics evaluated the contribution of each of the eye-tracking test sub-variables to the new model. Furthermore, multicollinearity was evaluated through the variation inflation factor (VIF). Alpha levels were set at a *p*-value of <0.05 for all analyses. The underlying formulas for testing psychometrics were previously described (Baeyens et al., 2019). A sample size of 53 participants is considered statistically adequate, assuming a medium-size relationship between the independent and dependent variables, with alpha = 0.05; therefore, a study-specific power analysis was not performed (Tabachnick and Fidell, 2001; Green, 1991).

## Results

### Participant demographics

The 53 participants were between the ages of 20 and 43 years (20.1 ± 5.7), with 25 of them being male. Of the 53 participants, 73% were white, 10% were Black, 12% were Hispanic, and 5% opted not to report ethnicity. One individual who provided consent was excluded from the study during pre-screening due to recent alcohol consumption, and one participant experienced a problematic Internet connection during the eye-tracking tests, which led to their exclusion from some regression analyses. The prevalence of an abnormal oculomotor function, as classified by the neurologist, ranged from 50.0% (HS) to 59.6% (HRSs, CSPs, and HSPs).

### Test order

**Figure 2:**
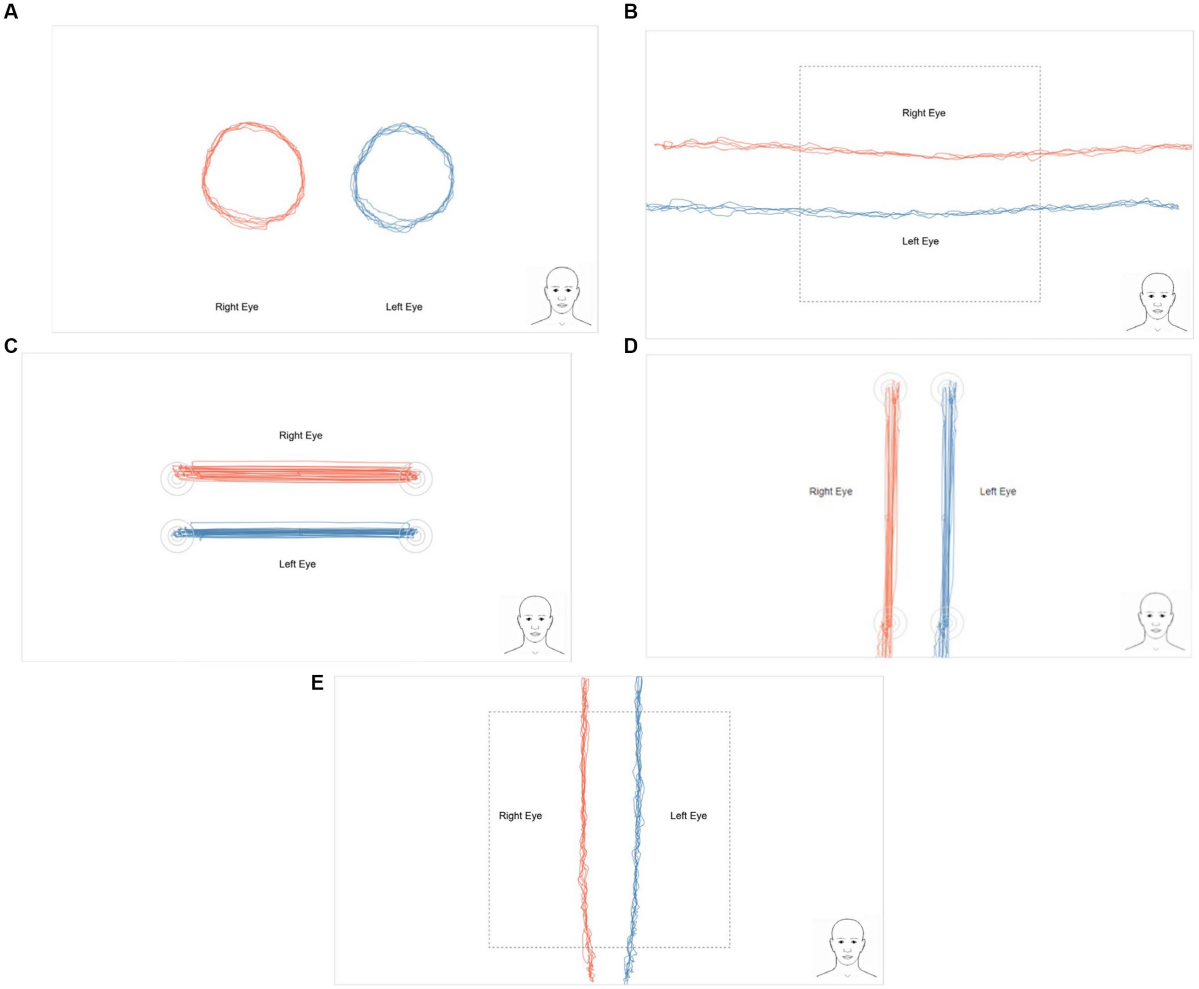
Sample eye traces for each of the RightEye tests performed by an uninjured participant: **(A)** circular smooth pursuits, **(B)** horizontal smooth pursuits, **(C)** vertical smooth pursuits, **(D)** horizontal saccades, and **(E)** vertical saccades. Horizontal random saccade traces are not recorded.

All variables produced a non-significant result for order (*p* > 0.05). As such, there was no difference in whether a participant received computerized eye-tracking first or second. Figure 2 provides sample eye traces for five computerized eye-tracking tests (HRSs did not capture traces). In addition, multicollinearity was evaluated for each model, and it was determined for all models and variables to be well below 10 or a value that would indicate severe multicollinearity.

### Horizontal random saccade test

The HRS logistic regression examined the concurrent validity of the 16 HRS test metrics against the clinician-rated classification of oculomotor function. The Hosmer–Lemeshow test results (*p* = 0.94) suggested that the model was well-fit. The full model of 16 predictor variables significantly predicted clinically evaluated HRS status (*χ^2^* = 51.730, *df* = 16, *n* = 52, *p* < 0.001) (Table 1). There were no significant individual predictors (Wald statistic *p* > 0.05). The HRS variables explained 89% of the variance in the model, and 92.3% of individuals were correctly predicted (i.e., overall model accuracy). Sensitivity and specificity were 93.5% and 90.5%, respectively.

**Table 1:** Horizontal random saccade logistic regression model summary.

HRS model variables	β	SE	VIF	95% CI for EXP(β)	
				Lower	Upper
Constant	67.84	121.4			
0 degrees velocity fast phase	0.231	0.254	4.198	0.57	1.35
0 degrees velocity slow phase	−0.451	0.134	3.067	0.41	2.37
0 degrees duration fast phase	2.876	0.381	1.633	131.95	227.79
0 degrees duration slow phase	0.190	1.130	1.303	60.01	220.69
20 degrees velocity right fast phase	0.347	0.329	3.144	0.667	3.067
20 degrees velocity right slow phase	1.581	0.811	3.492	2.311	8.091
20 degrees duration right fast phase	−0.135	0.219	1.580	257.9	549.66
20 degrees duration right slow phase	−1.981	0.378	2.241	411.72	586.26
20 degrees velocity left fast phase	2.879	0.561	1.925	0.89	3.87
20 degrees velocity left slow phase	3.271	0.692	3.884	3.46	8.54
20 degrees duration left fast phase	−0.129	0.382	3.636	233.94	474.52
20 degrees duration left slow phase	0.329	0.562	1.315	320.67	682.18
23 degrees velocity left fast phase	−0.189	0.768	2.593	1.72	3.74
23 degrees velocity left slow phase	0.970	0.689	3.643	3.65	8.99
23 degrees duration left fast phase	1.289	0.349	2.095	206.13	567.23
23 degrees duration left slow phase	−2.349	0.568	3.441	320.02	615.76

HRSs, horizontal random saccades; SE, standard error; VIF, variation inflation factor; CI, confidence interval.

### Circular smooth pursuit test

The CSP logistic regression examined the concurrent validity of the five CSP test metrics against the corresponding clinician-administered oculomotor test. The Hosmer–Lemeshow test results (*p* = 0.94) suggested that the model was well-fit. The full model of the five predictor variables significantly predicted clinically evaluated CSP status (*χ^2^* = 52.640, *df* = 5, *n* = 52, *p* < 0.001) (Table 2). There were no significant individual predictors (Wald statistic *p* > 0.05). The CSP variables explained 86% of the variance in the model, and 92.3% of individuals were correctly predicted (i.e., overall model accuracy). Sensitivity and specificity were 93.5% and 90.5%, respectively.

**Table 2:** Circular smooth pursuit logistic regression model summary.

CSP model variables	β	SE	VIF	95% CI for EXP(β)	
				Lower	Upper
Constant	23.718	64.496			
CSP fixation (%)	−0.902	0.780	2.824	0.088	1.872
CSP latent SP (%)	−0.074	0.157	2.262	0.683	1.263
CSP On-target SP (%)	0.027	0.170	4.058	0.736	1.435
CSP SP (%)	−0.617	0.569	3.337	0.177	1.646
CSP E/T VE (°)	2.418	1.718	4.816	0.387	325.322

CSPs, circular smooth pursuits; SE, standard error; VIF, variation inflation factor; CI, confidence interval; SP, smooth pursuits; E/T VE (°), eye target travel velocity.

### Horizontal smooth pursuit test

The HSP logistic regression examined the concurrent validity of the four HSP test metrics against the corresponding clinician-administered oculomotor test. The Hosmer–Lemeshow test results (*p* = 0.78) suggested that the model was well-fit. The full model with four predictor variables significantly predicted and clinically evaluated the HSP status (*χ^2^* = 25.402, *df* = 4, *n* = 52, *p* < 0.001) (Table 3). HSP SP (%) was the only significant individual predictor (Wald statistic *p* = 0.043). The HSP variables explained 52% of the variance in the model, and 92.3% of individuals were correctly predicted (i.e., overall model accuracy). Sensitivity and specificity were 93.5% and 90.5%, respectively.

**Table 3:** Horizontal smooth pursuit logistic regression model summary.

HSP model variables	β	SE	VIF	95% CI for EXP(*β*)	
				Lower	Upper
Constant	15.784	12.262			
HSP blink (#)	−0.103	0.134	1.699	0.694	1.172
HSP E/T VE (°)	0.356	0.325	1.777	0.755	2.700
HSP saccade (%)	−0.030	0.309	1.086	0.529	1.778
HSP SP (%)^*^	−0.236	0.117	2.194	0.628	0.993

HSPs, horizontal smooth pursuits; SE, standard error; VIF, variation inflation factor; CI, confidence interval; E/T VE (°), eye target travel velocity; SP, smooth pursuits. ^*^p < 0.05.

### Vertical smooth pursuit test

The VSP test regression analysis examined the concurrent validity of the five VSP test metrics against the corresponding clinician-administered oculomotor test. The Hosmer–Lemeshow test results (*p* = 0.33) suggested that the model was a good fit. The full model of the five predictor variables significantly predicted clinically evaluated VSP status (*χ^2^* = 30.347, *df* = 5, *n* = 53, *p* < 0.001) (Table 4). VSP sync-y was the only significant individual predictor (Wald statistic *p* = 0.017). The VSP variables explained 58% of the variance in the model, and 86.8% of individuals were correctly predicted (i.e., overall model accuracy). Sensitivity and specificity were 88.9% and 84.6%, respectively.

**Table 4:** Vertical smooth pursuit logistic regression model summary.

VSP model variables	β	SE	VIF	95% CI for EXP(β)	
				Lower	Upper
Constant	9.810	7.462			
VSP blink (#)	0.107	0.148	1.310	0.833	1.488
VSP E/T VR (°)	−0.007	0.097	4.746	0.820	1.201
VSP fixation (%)	−0.010	0.073	1.301	0.858	1.141
VSP SP (%)	−0.062	0.089	1.153	0.789	1.120
VSP sync-y^*^	−7.703	3.225	1.017	0.000	0.251

VSPs, vertical smooth pursuits; SE, standard error; VIF, variation inflation factor; CI, confidence interval; E/T VR (°), eye target travel velocity. ^*^p < 0.05.

### Horizontal saccade test

The HS test regression examined the concurrent validity of the four HS test metrics against the corresponding clinician-administered oculomotor test. The Hosmer–Lemeshow test results (*p* = 0.24) suggested that the model was a good fit. The full model of four predictor variables significantly predicted clinically evaluated HS status (*χ^2^* = 25.523, *df* = 4, *n* = 52, *p* < 0.001) (Table 5). HS All (#) was the only significant individual predictor (Wald statistic *p* < 0.001). The HS variables explained 52% of the variance in the model, and 84.6% of individuals were correctly predicted (i.e., overall model accuracy). Sensitivity and specificity were 84.6% and 87.4%, respectively.

**Table 5:** Horizontal saccade logistic regression model summary.

HS model variables	β	SE	VIF	95% CI for EXP(β)	
				Lower	Upper
Constant	0.718	0.932			
HS fixation (#)	−0.531	0.596	1.108	0.183	1.891
HS on-target (#)	0.166	0.137	1.164	0.902	1.544
HS saccade (#)	0.258	0.575	1.248	0.419	3.994
HS All (#)^**^	0.504	0.154	1.461	1.223	2.240

HSs, horizontal saccades; SE, standard error; VIF, variation inflation factor; CI, confidence interval. ^**^p < 0.01.

### Vertical saccade test

The VS test regression examined the concurrent validity of the five VS test metrics against the corresponding clinician-administered oculomotor test. The Hosmer–Lemeshow test results (*p* = 0.68) suggested that the model was well-fit (Table 6). The full model of the five predictor variables significantly predicted clinically evaluated VS status (*χ^2^* = 19.351, *df* = 5, *n* = 52, *p* < 0.01). VS All (#) was the only significant individual predictor (Wald statistic *p* = 0.007). The VS variables explained 42% of the variance in the model, and 78.8% of individuals were correctly predicted (i.e., overall model accuracy). Sensitivity and specificity were 70.4% and 88.0%, respectively.

**Table 6:** Vertical saccade logistic regression model summary.

VS model	β	SE	VIF	95% CI for EXP(β)	
				Lower	Upper
Constant	0.036	1.284			
VS fixation (#)	0.467	0.491	1.126	0.610	4.174
VS on-target (#)	−0.023	0.104	1.047	0.797	1.197
VS fixation (%)	0.017	0.056	1.053	0.911	1.136
VS saccade (#)	−0.550	0.470	1.121	0.230	1.449
VS All (#)^**^	0.492	0.182	1.671	1.145	2.338

VSs, vertical saccades; SE, standard error; VIF, variation inflation factor; CI, confidence interval. ^**^p < 0.01.

**Figure 3:**
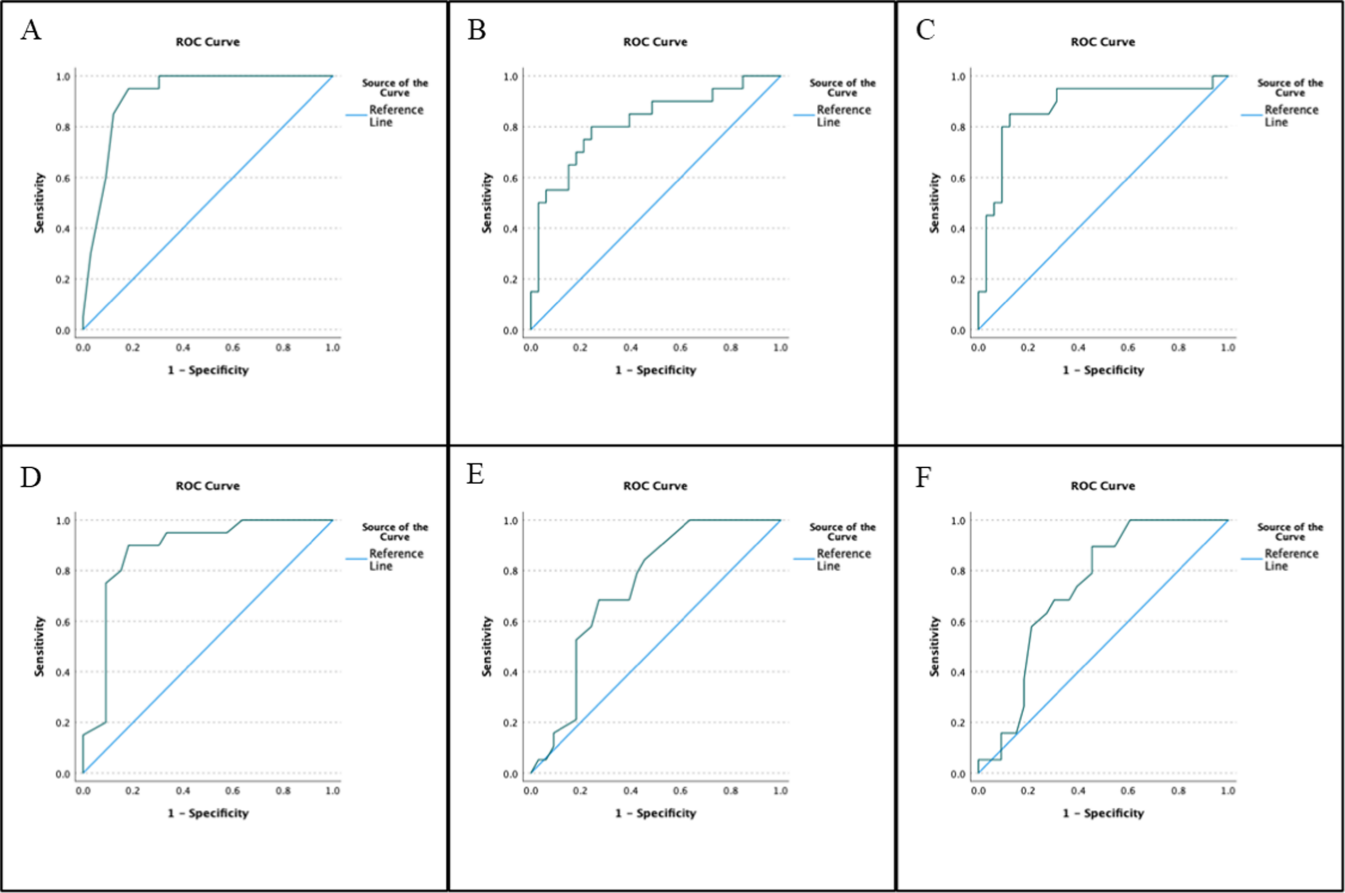
RightEye tests summary ROC matrix for the following oculomotor tests: **(A)** horizontal random saccades, **(B)** circular smooth pursuits, **(C)** horizontal smooth pursuits, **(D)** vertical smooth pursuits, **(E)** horizontal saccades, and **(F)** vertical saccades.

Expanded diagnostic metrics for each eye-tracking test are presented in Table 7, and a summary of the ROC curves is shown in Figure 3.

**Table 7:** Psychometric summary table.

Test	TP	FN	FP	TN	LR+	LR-	Accuracy	Sn	Sp	PPV	NPV	Youden’s J	AUC	DOR
HRS	29	2	2	19	9.82	0.07	92.3%	93.5%	90.5%	0.94	0.90	0.84	0.921	137.8
CSP	29	2	2	19	9.82	0.07	92.3%	93.5%	90.5%	0.94	0.90	0.84	0.817	137.8
HSP	29	2	2	19	9.82	0.07	92.3%	93.5%	90.5%	0.94	0.90	0.84	0.876	137.8
VSP	24	3	4	22	5.78	0.13	86.8%	88.9%	84.6%	0.86	0.88	0.74	0.879	44.0
HS	22	4	4	22	5.50	0.18	84.6%	84.6%	84.6%	0.85	0.85	0.69	0.735	30.3
VS	19	8	3	22	5.86	0.34	78.8%	70.4%	88.0%	0.86	0.73	0.58	0.734	17.4

TP, true positive; FN, false negative; FP, false positive; TN, true negative; LR+, positive likelihood ratio; LR−, negative likelihood ratio; SN, sensitivity; SP, specificity; PPV, positive predictive value; NPV, negative predictive value; AUC, area under the curve; DOR, diagnostic odds ratio; HRSs, horizontal random saccades; CSPs, circular smooth pursuits; HSPs, horizontal smooth pursuits; VSPs, vertical smooth pursuits; HSs, horizontal saccades; VSs, vertical saccades.

## Discussion

The purpose of this study was to assess the validity of a computerized eye-tracking apparatus by comparing the results of six computerized oculomotor tests (i.e., index tests) to the results of a clinician-administered oculomotor examination (both individual tests and overall performance) performed by a board-certified neurologist with 16 years of experience (i.e., reference standard). The clinician-administered oculomotor examination identified ≥50% of the participants as having an abnormal test. These rates are higher than previous studies involving college-aged participants (13.2%–34.2%) (García-Muñoz et al., 2016; Porcar and Martinez-Palomera, 1997; Badovinac et al., 2017) and healthy adults in their 50s (31%) (Leat et al., 2013), but comparable to those found in older adults ages 60 and above (41%–51%). All six eye-tracking tests independently predicted the classification of the corresponding oculomotor examination component or the overall classification determined by the neurologist. Sensitivity was highest in the HRS, CSP, and HSP tests (93.5%) and lowest in the HS tests (84.6%). Specificity was highest in the HRS, CSP, and HSP tests (90.5%) and lowest in the VS test (70.4%). Classification accuracy ranged from 78.8% (VS) to 92.3% (HRS, CSP, and HSP), and the AUC values ranged from 0.734 (VS) to 0.921 (HRS). Additionally, several individual test metrics independently predicted abnormal classification, including HSP SP, VSP sync-y, HS All (number), and VS All (number). The pursuit test demonstrated higher sensitivity and specificity, likely due to the type of test. In previous studies, pursuit tasks, in general, have demonstrated great sensitivity and specificity to differentiating populations’ neuro-dysfunction (e.g., mTBI), and our results align with this finding. It is important to mention that VOR, vergence, and OKR were not evaluated by the neurologist or by the computerized eye-tracking system. Although these are sometimes used as tests, we utilized oculomotor tests that, in our previous studies, distinguished these types of groups (Murray and Hunfalvay, 2017). The results suggest that each of the six computerized eye-tracking tests can accurately distinguish between normal and abnormal oculomotor movements. This study was not designed to determine the cause of abnormal movements.

These data represent the first evidence validating the computerized eye-tracking tests as clinically comparable to a board-certified neurologist’s oculomotor examination. This cohort reported being naïve to eye-tracking technology and was not under the influence of recreational drugs or alcohol, which likely reflects the expected target population for its intended use (i.e., clinical, office-based setting). We also believe that the random assignment of test order and the blinding of both the neurologist and researcher further strengthen the credibility of the results.

Neurologists, ophthalmologists, and optometrists may find that eye-tracking technologies offer three direct benefits to their clinical practice: (1) quantifying eye movements, (2) longitudinal tracking of a patient’s progress (or regression), and (3) standardizing patient evaluations to focus on treatment. Considering the results from this validation study in conjunction with the reliability and normative data previously published, (Murray and Hunfalvay, 2017) there is preliminary support for the computerized apparatus to be a tool for assessing saccades and smooth pursuits eye movements (excluding vergence, VOR, and OKR).

## Conclusion

While the researchers were thoughtful in the design and execution of the project, this study was not without limitations. The characteristics of the participants may represent a limitation. Due to the unknown etiology of abnormal eye movements, we were unable to determine whether certain diagnosed or undiagnosed neurological diseases may have affected the accuracy of the computerized apparatus. Furthermore, the use of a single clinician and the study’s design are possible limitations. Although it is standard practice and the current ‘gold standard’ to have a single clinician perform these tests, it is possible that the conclusions derived by the clinician, even with 16 years of clinical practice, may have limitations. Eye-tracking has the potential to add value by contributing to a more objective conclusion. Another challenge was the relatively small sample size; however, a *post-hoc* power analysis indicated that the study was sufficiently powered. Furthermore, with the participants aged 20–43 years old, these results should not be generalized to all ages of the lifespan (e.g., minors or older adults). Additionally, the age range was chosen to reduce the opportunity for the results to be influenced by age. Importantly, the testing was described as an oculomotor exam, but we acknowledge that it was not a comprehensive assessment of the oculomotor system (VOR, OKR, and vergence excluded). HRSs, as an overall measure of oculomotor function, require further investigation to determine if the computerized test performance is predictive of VOR, OKR, or vergence testing results. Therefore, the results of HRS diagnostic performance should not be applied generally, and the “overall oculomotor function” must be framed within the context that VOR, OKR, and vergence testing were not analyzed in this study. As technological advances are introduced to the medical community, clinicians must learn how to incorporate biotechnology into their practice when appropriate. Leveraging technology may improve diagnostic capabilities, reduce inefficiencies, and enhance clinical care.

## Data Availability

The raw data supporting the conclusions of this article will be made available by the authors, without undue reservation.
